# Post-event follow-up costs in patients with atherosclerotic cardiovascular disease in Spain

**DOI:** 10.3389/fcvm.2024.1324537

**Published:** 2024-02-28

**Authors:** Icíar Martínez López, Jorge Francisco Gómez Cerezo, José M. Gámez, Isabel Egocheaga Cabello, Mar Castellanos, Raquel Campuzano Ruiz, Vivencio Barrios, Vicente Pallarés-Carratalá, José Manuel Rodríguez, Nuria Morant Talamante, Javier Parrondo, José María Mostaza

**Affiliations:** ^1^ Pharmacy Service and Molecular Diagnostics and Clinical Genetics Unit, Hospital Universitario Son Espases, Balearic Islands, Palma de Mallorca, Spain; ^2^ Internal Medicine Service, Hospital Universitario Infanta Sofía, Universidad Europea de Madrid, Madrid, Spain; ^3^ Cardiology Service, Hospital Universitario Son Llatzer, Palma de Mallorca, Spain; ^4^ Department of Medicine, Universidad de las Islas Baleares, Palma de Mallorca, Spain; ^5^ CIBER of Physiopathology of Obesity and Nutrition (CIBEROBN CB 12/03/30038), Instituto de Salud Carlos III, Madrid, Spain; ^6^ Family Medicine Service, Health Center of Isla de Oza, Madrid, Spain; ^7^ Department of Neurology, University Hospital and Biomedical Research Institute, A Coruña, Spain; ^8^ Cardiac Rehabilitation, Cardiology Service, Hospital Universitario Fundación Alcorcón, Madrid, Spain; ^9^ Hospital Universitario Ramón y Cajal and Medicine and Medical Specialties Department, Universidad de Alcalá de Henares, Madrid, Spain; ^10^ Health Surveillance Unit, Unión de Mutuas, Castellón, Spain; ^11^ Medicine Department, Universitat Jaume I, Castellón, Spain; ^12^ Health Economics, Novartis Pharmaceuticals, Madrid, Spain; ^13^ Medical Department, Novartis Pharmaceuticals, Barcelona, Spain; ^14^ Internal Medicine Section, Hospital Universitario la Paz, Madrid, Spain

**Keywords:** cost of illness, hypercholesterolemia, atherosclerosis, observational study, electronic health records

## Abstract

**Introduction:**

Atherosclerotic cardiovascular disease (ASCVD) is one of the main causes of morbidity and mortality in developed countries and entails high resources use and costs for health systems. The risk of suffering future cardiovascular (CV) events and the consequent resources use is higher in those patients who have already had a previous cardiovascular event. The objective of the study was to determine the average annual cost of patients with a new or recurrent atherosclerotic CV event during the 2 years after the event.

**Methodology:**

Retrospective observational study of electronic medical records of patients from the BIG-PAC® database (7 integrated health areas of 7 Autonomous Communities; *n* = 1.8 million). Patients with a new or recurrent episode of ASCVD (angina, acute myocardial infarction, transient ischemic attack, stroke, or peripheral arterial disease) between 1-Jan-2017 and 31-Dec-2018 were included. The resources use within two years of the diagnosis was estimated in order to estimate the average cost of patient follow-up.

**Results:**

A total of 26,976 patients with an ASCVD episode were identified during the recruitment period; Out of them, 6,798 had a recurrent event during the follow-up period and 2,414 died. The average costs per patient were €11,171 during the first year and €9,944 during the second year.

**Discussion:**

Patients with ASCVD represent a significant economic burden for the health system and for society. Despite the perception that drug costs in the follow-up of chronic patients imply a high percentage of the costs, these accounted for only one tenth of the total amount. Implementing preventive programs and increasing the control of cardiovascular risk factors may have a significant social and health impact by helping to reduce mortality and costs for the Spanish National Health System. The costs derived from pharmacological treatments were obtained from the NHS pricing nomenclator database (https://www.sanidad.gob.es/profesionales/nomenclator.do).

## Introduction

Cardiovascular (CV) disease is the main cause of mortality in the world, representing 32% of global deaths with 17.9 million deaths in 2019 (1). Specifically, atherosclerotic cardiovascular disease (ASCVD) is the main cause of death with ischemic heart disease and stroke being the first two causes of death in the world with 16% and 11% of recorded mortality (2). In addition to mortality, ASCVD entails a high morbidity and high resources use and costs for health systems (1, 3, 4).

In upper-middle and high-income countries, ASCVD is one of the leading causes of mortality. Despite decreasing deaths from ischaemic heart disease and stroke between 2000 and 2019, they remain the first and third most common cause of death respectively, contributing to a total of 2.5 million death in 2019 (2).

In Spain, cardiovascular disease affects 9.8% of the population (52.6% of women and 47.4% of men) with an annual incidence in 2019 of one new case per 100 inhabitants, being the leading cause of death (27.9% of deaths) and hospital admissions (12.6%) (3).

LDL-cholesterol (LDL-C) is considered a causative agent of atherosclerosis with its pathophysiological mechanism of action being related to its infiltration and deposition in the vascular wall (5). Other factors that increase the risk of cardiovascular disease are smoking, diabetes, arterial hypertension, obesity, sedentary lifestyle and pollution (6, 7). Plasma LDL-C is one of the risk factors that can be modified, and its decrease reduces the rate of atherosclerotic CV events (8).

The risk of suffering CV complications in the future, with the consequent resources use, is higher in those patients who have suffered a previous cardiovascular event (3, 4, 7). In order to reduce this risk, it is necessary to implement secondary prevention programs based on the change of habits to healthier behaviors and on the establishment of drug treatments among which lipid-lowering therapy plays a key role (3).

Although the efficacy of lipid-lowering therapies are well established, their effectiveness in real-world clinical practice will depend on their appropriate prescription (and optimization) and on patient's adherence to therapy. This variability will affect both the clinical results and the resources use for the follow-up and treatment of patients.

The effectiveness data, the direct medical costs data estimated from the resources use, and the inclusion of other costs (e.g., indirect medical costs, non-medical costs) may be key factors for health authorities in the decision-making process (9).

In Spain, there is scarce evidence available of the administration of lipid-lowering therapies in clinical practice, their effectiveness, and the associated costs in patients with ASCVD and hypercholesterolemia (10).

The objective of this study was to assess the resource use by ASCVD patients (grouped and divided by the type of ASCVD on the index date) during a two-year follow-up period by estimating the cost during this follow-up.

## Methodology

A retrospective observational study was conducted using electronic medical records (EMR) from the BIG-PAC administrative database. This is a secondary data source owned by Atrys Health-RLD (http://www.encepp.eu/encepp/viewResource.htm?id = 29236#) that has been used for several studies of different pathologies (11–14). This includes anonymized data from electronic medical records of a population of 1.8 million patients of seven integrated health areas of the public health system, belonging to seven autonomous communities of Spain. These integrated areas include both primary care centers and hospitals and are a representative sample of the Spanish population (15).

**Figure 1:**
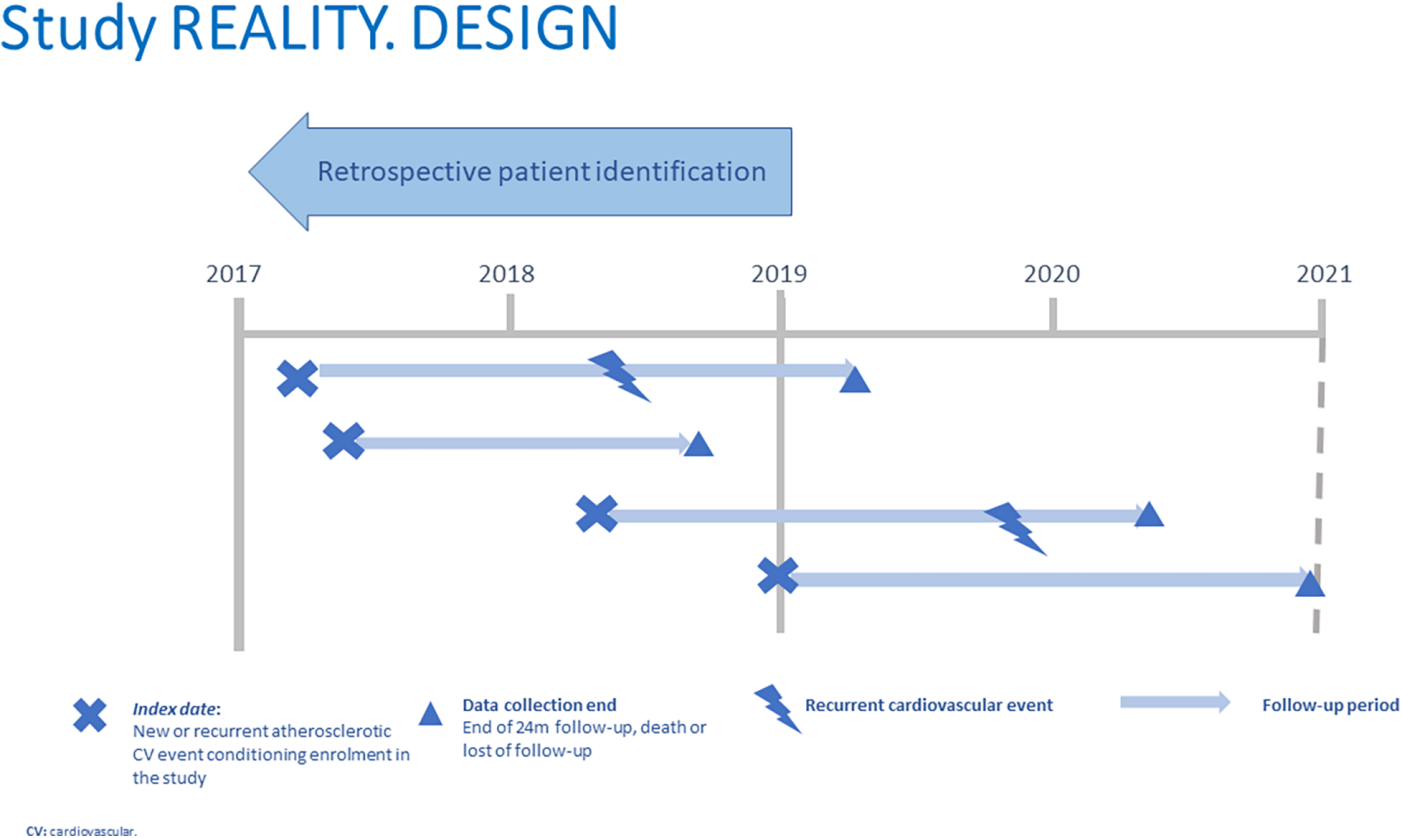
Study design.

The study protocol, including the inclusion and exclusion criteria, was the subject of a previous publication (16). Patients who during the identification period (between 1-Jan-2017 and 31-Dec-2018) had a new or recurrent ASCVD episode were enrolled in the study, considering the date of that episode as the index date from which a follow-up period of 24 months started (until 31-Dec-2020 at the latest). The study design is shown in Figure 1.

The ASCVD diagnosis was based on the following ICD-9-CM disease codes: Stable/unstable angina (413, 411); acute myocardial infarction (AMI) (410, 412); ischemic stroke (433–434, 436); transient ischemic attack (TIA) (435); and peripheral arterial disease (PAD) (440–441, 444). The ASCVD episode on the index date and the recurrent ASCVD events during the follow-up period were both diagnosed based on that classification. The criteria for the diagnosis of the health conditions were clinical, at the treating physician's discretion, and were based on the presence of signs/symptoms, as well as on findings of a structural/functional abnormality in the heart/vessels according to the result of the diagnostic tests performed. Due to the administrative nature of the BIGPAC database, the mortality data collected are all-cause mortality.

In order to determine the health resource use, including medical visits, diagnostic tests, and medication, were quantified during the 24-monthfollow-up, death or loss of follow-up.

Visits included primary care visits, outpatient department visits (cardiology, vascular surgery, endocrinology, geriatrics, internal, neurology and rehabilitation), emergency room visits and hospital admissions (number of hospital stays and days of hospital stay).

Diagnostic tests included laboratory tests (blood tests), conventional x-rays, computerized axial tomography (CAT) scans, magnetic resonance imaging (MRI), echocardiograms, stress tests, and Holter monitoring. And therapeutic procedures included catheterization, angioplasty, bypass graft, endarterectomy, thrombectomy and rehabilitation therapy.

Drug use data were obtained from drug dispensing logs, including lipid-lowering therapies and concomitant medication (such as antidiabetic, antithrombotic and antihypertensive agents, other agents acting on the renin-angiotensin system, non-steroidal anti-inflammatory drugs, and antiulcer agents/mucosal protective agents).

For the statistical analysis, these variables were described as mean, standard deviation and relative frequencies (%). The statistical software SPSS v 27 (SPSS Inc. Chicago, Illinois, USA) was used for the analysis.

Regarding the assessment of costs, direct medical costs and indirect costs were considered.

### Direct healthcare costs

Costs were allocated by multiplying the resources use by the unit cost of each resource. These unit costs are shown in Table 1 and have been estimated based on the average official fees of the different autonomous communities. Assuming that these are the fees used by the regional health systems for charging the mutual societies and insurance companies for the procedures as an acceptable approximation to the willingness to pay by the Spanish National Health System. Fees were obtained thorough a search of the most recent official fees of the 17 autonomous communities (Supplementary Table S1 in the Appendix to this article). These were updated to the value of euros in 2021 using the tool “Update a personal income” of the Spanish National Statistics Institute (www.ine.es), and the national unit cost was determined by estimating the mean unit cost of all the autonomous communities. The costs derived from pharmacological treatments were obtained from the NHS pricing nomenclator database (https://www.sanidad.gob.es/profesionales/nomenclator.do). As the study seeks to estimate the average follow up cost per patient, the drug cost will be estimated from the pharmacies drug withdrawal regardless of whether the patient is adherent or not to medication.

**Table 1:** List of unit costs.

Concept	Cost €^a^
Primary care visit	49.17
Outpatient department visit	101.90
Emergency room visit	258.92
Days of hospital stay	631.54
Therapeutic procedures^b^	4,483.17
Blood test	13.49
Conventional x—ray	21.47
Computerized Axial Tomography (CAT)	240.53
Magnetic Resonance Imaging (MRI)	315.19
Other diagnostic procedures^c^	154.00
Cost for day of absence from work^d^	101.21

^a^ Average cost considering the mean of the published official tariffs inflated to €2021.

^b^ It includes angioplasty, endarterectomy, bypass, thrombectomy and rehabilitation.

^c^ It includes echocardiogram, stress test and Holter monitoring.

^d^ Source: Spanish National Statistics Institute.

### Indirect costs

Non-medical costs included costs associated with productivity loss; therefore, only the productivity loss in the working-age population (active population defined as those under 65 years of age) was computed. In order to measure said productivity loss, both the productivity loss due to days of absence from work (days off work) due to temporary or permanent disability and the productivity loss due to days of absence from work due to premature mortality were estimated.

In order to estimate the productivity loss due to days off work, the total number of days of absence from work due to temporary or permanent disability was multiplied by the average daily wage of the Spanish population.

Regarding the productivity loss due to premature mortality, the days lost were estimated subtracting the age of death from the retirement age in Spain (set at 65 years for the study). Costs were allocated by multiplying these days by the average daily wage of the Spanish population.

The average daily wage was estimated by dividing the average gross yearly wage by gender of the Spanish population by 365 days per year (17); updated to the value of euros in 2021.

The final results, the sum of the medical and non-medical costs considered, pooled by diagnosis of ASCVD were presented as average cost per patient in the value of euros in 2021.

## Results

A total of 26,976 patients who had had an atherosclerotic CV event during the identification period was identified [59.4% men; 69.9 years old (SD 11.5)]; 25.2% of them (6,798) suffered at least one recurrent atherosclerotic CV event, and 8, 9% of the patients enrrolled (2,414) died during the follow-up by type of ASCVD. The tables show the patient distribution and mean days of follow up (Table 2) and the recurring events and deaths recorded during the follow-up (Table 3) by type of ASCVD (angina, AMI, ischemic stroke, TIA, and PAD) on the index date. Table 4 summarizes the use of lipid-lowering drugs at the time of enrollment in the study, classified according to the consensus document of the Spanish Society of Cardiology (18). Drug treatments account for 11% of the average cost of the follow-up of these patients; it should be noted that only 0.9% of patients received protein convertase subtilisin/kexin type 9 inhibitors (PCSK9i).

**Table 2:** Number of patients, sex, age and follow-up per index ASCVD event.

	Angina	AMI^a^	STROKE^b^	TIA^c^	PAD^d^
Patients, *n*	6,794	5,556	6,388	2,184	6,054
Males (%)	63.0	59.8	53.3	49.0	65.3
Age, mean (SD)^e^	69.6 (11.5)	68.5 (12.3)	71.2 (11.8)	71.5 (11.2)	69.6 (10.3)
Days of follow-up mean (SD)	539 (230)	556 (228)	562 (226)	567 (224)	552 (228)

^a^ Acute myocardial infarction.

^b^ Ischemic stroke.

^c^ Transient ischemic attack.

^d^ Peripheral arterial disease.

^e^ Age at the index date.

**Table 3:** Number of patients with a recurrent ASCVD event in the follow-up and mortality per type of index ASCVD^a^.

	Angina	AMI^b^	STROKE^c^	TIA^d^	PAD^e^
Recurrent ASCVD^f^ (*n*)	2,134	1,378	1,220	450	1,614
Recurrent ASCVD^f^ (%)	31.4	24.8	19.1	20.6	26.7
Mortality^g^ (*n*)	500	298	760	238	618
Mortality^g^ (%)	7.4	5.4	11.9	10.9	10.2

^a^ The recurrent ASCVD event can be of the same type than the index event or of a different type.

^b^ Acute myocardial infarction.

^c^ Ischemic stroke.

^d^ Transient ischemic attack.

^e^ Peripheral arterial disease.

^f^ Percentage of patients who suffer an ASCVD during the follow-up period.

^g^ Percentage of patients who die for any cause during the follow-up.

**Table 4:** Use of lipid-lowering drugs by treatment intensity at the time of enrollment in the study.

Intensity	Description	Patients [*n* (%)]
		*N* = 26,976
Extreme	• Optimized treatment^a^ + PCSK9i	234 (0.9%)
Very high	• High-intensity statins^b^ + ezetimibe	9,831 (36.4%)
High	• High-intensity statins (alone)	14,285 (53%)
	• Moderate-intensity statins^b^ + ezetimibe	
Moderate	• Moderate-intensity statins	1,957 (7.3%)
	• Low-intensity statins^b^	
	• Low-intensity statins + ezetimibe	
Non-statins	• Other non-statin lipid-lowering drugs	669 (2.5%)

^a^ Optimized treatment + PCSK9i: protein convertase subtilisin/kexin type 9 inhibitor added to the treatment with statins at the maximum tolerated dose +/− ezetimibe.

^b^ Classification according to the consensus document of the Spanish Society of Cardiology (18).

Resource use in patient with ASCVD is shown in Table 5. (Resource use by type of ASCVD is shown in Supplementary Tables S2–S6 of the Appendix).

**Table 5:** Resource use for patients with ASCVD^a^.

	Year 1 follow-up (*n* = 26,976)		Year 2 follow-up (*n* = 25,744)	
	% of patients	$\bar{\times }$ per patient	% of patients	$\bar{\times }$ per patient
Visits and hospital stays				
Primary care visits	100.0	14.9	100.0	13.0
Outpatient department visits^b^	86.5	3.2	87.5	2.8
Emergency room	68.2	1.1	63.0	1.0
Days of hospital stay	13.7	2.1	11.5	1.8
Therapeutic procedures^c^	57.6	0.9	31.2	0.9
Diagnostic te sts				
Blood tests	80.2	2.4	67.6	2.2
Conventional x-ray	25.7	0.5	21.4	0.6
Computerized Axial Tomography	54.3	0.8	53.0	0.7
Magnetic Resonance Imaging	58.8	0.7	31.6	0.5
Other diagnostic procedures^d^	71.6	3.1	53.3	2.4
Productivity loss (Indirect costs)				
Temporary disability (days off work)	12.8	12.8	10.1	10.7
Permanent disability	1.1	—	1.2	—
Premature mortality	0.4	—	0.4	—

^a^ Percentage of patients who use resources and mean of patients within the total study population.

^b^ Cardiology, vascular surgery, endocrinology, geriatrics, internal medicine, neurology and/or rehabilitation.

^c^ Catheterization, angioplasty, bypass, endarterectomy, thrombectomy and rehabilitation therapy.

^d^ Echocardiogram, stress test and Holter monitoring.

The estimated average cost per ACSVD patient and its different components (visits and hospital stays, diagnostic test, Drug treatments and productivity loss) is shown in Table 6 (additionally Supplementary Tables S7–S11 in the Appendix show these data by type of ASCVD. Overall, the estimated cost of follow-up in the first year was €11,171 (direct medical costs €9,021; indirect costs €2,150) and €9,944 (direct medical costs €7,839; non-medical costs €2,105) in the second year in patients with ASCVD. Table 7 shows the breakdown of average (medical and non-medical) costs per patient for each type of ASCVD.

**Table 6:** Follow-up costs per patient with ASCVD [mean (SD)] in the value of euros in 2021.

	Year 1 (*n* = 26,976)	Year 2 (*n* = 25,744)
Visits and hospital stays		
Primary care visits	731 (548)	363 (291)
Outpatient department visits^a^	327 (357)	284 (341)
Emergency room	285 (367)	251 (295)
Days of hospital stay	4,203 (7,754)	3,954 (6,494)
Therapeutic procedures^b^	1,313 (3,873)	1,149 (3,636)
Diagnostic tests		
Blood tests	33 (30)	29 (32)
Conventional x-ray	10 (18)	13 (29)
Computerized Axial Tomography	181 (208)	170 (198)
Magnetic Resonance Imaging	220 (209)	149 (248)
Other diagnostic procedures^c^	482 (256)	367 (222)
Drug treatments	1,236 (585)	1,111 (546)
Productivity loss (Indirect costs)		
Temporary disability	1,233 (3,753)	1,078 (4,662)
Permanent disability	423 (3,888)	432 (3,967)
Premature mortality	494 (11,106)	595 (12,877)

^a^ Cardiology, vascular surgery, endocrinology, geriatrics, internal medicine, neurology and/or rehabilitation.

^b^ Catheterization, angioplasty, bypass, endarterectomy, thrombectomy and rehabilitation therapy.

^c^ Echocardiogram, stress test and Holter monitoring.

**Table 7:** Breakdown of average costs per patient and per type of ASCVD.

	Year 1 (the value of euros in 2021)	Year 2 (the value of euros in 2021)
Angina		
Direct medical costs	9,512	8,566
Indirect costs	1,967	2,277
Total annual cost	11,479	10,843
Total (2-year) follow-up cost	22,322	
Acute myocardial infarction		
Direct medical costs	8,679	7,548
Indirect costs	1,961	2,114
Total annual cost	10,640	9,662
Total (2-year) follow-up cost	20,302	
Ischemic stroke		
Direct medical costs	8,778	7,141
Indirect costs	2,217	1,946
Total annual cost	10,995	9,087
Total (2-year) follow-up cost	20,082	
Transient ischemic attack		
Direct medical costs	8,901	7,644
Indirect costs	1,582	1,154
Total annual cost	10,483	8,798
Total (2-year) follow-up cost	19,281	
Peripheral arterial disease		
Direct medical costs	9,082	8,098
Indirect costs	2,663	2,415
Total annual cost	11,745	10,513
Total (2-year) follow-up cost	22,258	

## Discussion

The results of the study confirm that ASCVD patients have a high rate of recurrence of atherosclerotic CV events and short-term mortality. Almost one in ten patients in the study died during the two years of follow-up and one out of four suffered a new atherosclerotic CV event. This resulted in an average cost per patient of more than €10,000 per year which, together with the high prevalence of this disease, represents an important economic burden for the health system; this result confirms the findings of previous studies (3, 4).

Regarding the components of the follow-up cost, the highest cost derived from hospital admissions, a finding consistent with the high number of events, followed by costs derived from therapeutic procedures, drugs and leaves due to temporary disability in active patients.

Regarding the data broken down by type of ASCVD, Table 2 shows that the highest mortality occurred in patients whose index event was cerebrovascular (ischemic STROKE 11.2% and AIT 10.9%) and the lowest mortality occurred in patients with coronary disease (5.4% in AMI and 7.4% in angina). It should be noted that PAD (10.2%) was the second cause of mortality after cerebrovascular pathologies. The presence of a higher cerebrovascular mortality as compared to coronary mortality could be paradoxical if we consider the number of recurrent atherosclerotic CV events, which is higher in patients with coronary disease (19.1% recurrence in stroke vs. 24.8% in AMI and 31.4% in angina). This fact could have several possible explanations. Recurrent events in coronary disease may be more common, but mortality is lower, mainly in the case of angina episodes. It may also be due to the fact that the intensive lipid-lowering therapy had less impact in the secondary prevention of stroke as compared to coronary disease. On the other hand, it could be due to the fact that patients with a residual stroke have many complications that reduce their life expectancy, such as swallowing disorders, infections, etc. It may also be due to the shorter treatment window period in the stroke code as compared to the infarction code, or because the stroke code is not activated in patients with significant sequelae of a previous stroke (as specified in the activation criteria), which does not occur with the Code Infarction (19, 20). In any case, although both mortality from coronary disease and mortality from stroke have decreased significantly in recent years (2, 3), improvements in the secondary prevention of both are required, mainly in relation to the implementation of stroke units. The lower recurrence of stroke observed may be due to the increase in revascularization procedures in patients with stroke in cases in which severe carotid artery stenosis is detected. In these cases, the revascularization procedure is a high-impact therapeutic measure in the prevention of new recurrences. However, the higher mortality rate in patients with stroke may decrease the probability of recurrence.

With respect to the resources use, it is noteworthy that the results show that laboratory tests in the first year of follow-up were not recorded in the database in around 20% of patients with ASCVD and that this situation worsens during the second year in which this lack of control reaches 30%. This pattern is consistent in the different types of ASCVD analyzed with slight differences.

In this sense it should be noted that the 2019(21) and 2021 (7) European Society of Cardiology (ESC) Guidelines, the 2019 Joint European Society of Cardiology/European Atherosclerosis Society (ESC/EAS) Guidelines (22) and the European Stroke Organization (ESO) Guidelines (23) recommend to monitor the patient at least four to six weeks after the event followed by periodic annual follow-up. A recent study conducted with 346 patients in the Cádiz area shows that an initial close follow-up followed by an annual follow-up of lipid parameters after an acute coronary syndrome can help to optimize lipid-lowering therapy over three months, so that more than 80% of patients reach the therapeutic goals set in the guidelines (<55 mg/dl). This resulted in 4% of MACE events and 1.5% mortality per year (24, 25).

In comparison with other published studies, the €8,778 of direct medical costs in the first year in the ischemic stroke group are in line with the €8,623 estimated in the study CONOCES (26). Likewise, the €8,679 cost in the group of patients with AMI may be in line with the hospital cost of €7,185 in AMI described by Darba, et al*.* (27) considering that the latter does not include primary care costs (visits and medication) which in our study amounted to less than €2,000. These costs are also higher than those reported in the study conducted by Escobar, et al*.* (28). In this case, we observed that the costs used in this study are too low as compared to the willingness to pay using the official fees and that their description does not include substantial components such as therapeutic procedures, other diagnostic tests performed to these patients and the drug treatment they receive. All this implies an underestimation of the follow-up cost of these patients which can explain the differences with our study.

Data reveal higher direct medical costs in the follow-up of patients with angina as compared to the rest of the index pathologies. The main cost component were therapeutic procedures, accounting for an average cost of €3,211 in patients with angina; €2,523 in patients with PAD; €2,307 in patients with AMI; €2,055 in patients with TIA and €1,880 in patients with ischemic stroke. This is due to the fact that in the group of patients with angina as the index event, the percentage of patients who required therapeutic procedures and the average number of such procedures was significantly higher. These data contrast with the results of the CLARIFY registry, which shows a lower number of revascularization procedures performed in patients with stable angina. This finding could be due to the fact that in our study data from all patients with angina were pooled, including those with unstable angina (ACS), which may represent a limitation for the study.

As for other limitations of the study, those are specific limitations of retrospective observational studies when databases of an administrative nature such as BIG-PAC are used. Thus, underreporting due to inaccuracies in the diagnostic coding of the patients may occur in this type of studies. They may include incomplete follow-up data if, during the follow-up period, the study population was seen in other public or private facilities that are not part of its area of influence. Or it may happen that the database does not include some variables that could have an impact on the results of the study. In the case of primary care visits, the record does not allow us to discern whether the visit is a clinical visit or another of a bureaucratic nature (e.g., to collect a prescription). However, in this case there is no cost overestimation since the fee per visit is calculated with all types of medical acts (clinical and bureucratic). Another limitation of these databases is the lack of risk stratification due to the use of diagnostic coding and the loss of information that this entails.

Among the absent variables that could have an impact on the final economic results, the fact that study did not include data of patients attending cardiac rehabilitation programs or preventive programs should be considered. In addition, the estimation of indirect cost has been limited to the loss of productivity. This approach does not take into account the contributions that people aged over 65 make to society. Lastly, its main limitation is that the non-medical costs associated with patient care (formal and informal care) could not be considered. This type of social costs can be extremely significant when the atherosclerotic CV event results in severe disability. For example, this applies to the ischemic stroke; These costs reached €18,377 resulting in two thirds of the cost per patient in the first year in the CONOCES study (26). Other non-medical factors related to social and economic inequities are beyond this study and should be subject to future studies.

Despite these limitations, this is the first study to show a global picture of the follow-up costs of patients with ASCVD in Spain and their analysis can help decision-making for the management of this pathology and for the design of new studies that help to acquire knowledge about this pathology as well as help to determine how implementation of these data to the present time may have been affected by the recent COVID-19 epidemic.

## Conclusions

During the study period, which covers the years 2017 to 2020, the follow-up cost of patients who suffered an atherosclerotic CV event is significant, mainly due to hospitalization and therapeutic procedures costs. Despite the perception that drug costs in the follow-up of chronic patients imply a high percentage of the costs, these accounted for only one tenth of the total amount.

Improving the control and follow-up of patients through the development and implementation of preventive programs that help improve control of cardiovascular risk factors could help reduce their mortality and the cost for the Spanish National Health System.

## Data Availability

The data analyzed in this study is subject to the following licenses/restrictions: The BIG-PAC administrative database. This is a secondary data source owned by Atrys Health-RLD (http://www.encepp.eu/encepp/viewResource.htm?id=29236#). Requests to access these datasets should be directed to the BIG-PAC administrative database. This is a secondary data source owned by Atrys Health-RLD (http://www.encepp.eu/encepp/viewResource.htm?id=29236#).
