# Supplementary material for: Post-event follow-up costs in patients with atherosclerotic cardiovascular disease in Spain
Source: Front Cardiovasc Med. 2024 Feb 28;11:1324537. doi: 10.3389/fcvm.2024.1324537 (PMC10932982; doi:10.3389/fcvm.2024.1324537)
Supplement: Supplementary file 1 [file Table1.docx]

# Appendix: Supplementary data

Supplementary Table 1 List of official fees considered for the conduct of the study

| **Fee / Autonomous community** | **Source:** |
| --- | --- |
| Andalusia | Official Gazette of the Regional Government of Andalusia no. 210 27-Oct-2005 |
| Aragon | Official Gazette of Aragon no. 156 10-Aug-2012 |
| Asturias | Official Gazette of the Principality of Asturias no. 77 04-April-2013 |
| Balearic Islands | Official Gazette of the Balearic Islands no. 2 04-Jan-2018 |
| Canary Islands | Official Gazette of the Canary Islands no. 67 5-Apr-2017 |
| Cantabria | Official Gazette of Cantabria no. 248 29-Dec-2017 |
| Castilla La Mancha | Official Gazette of Castilla La Mancha no. 226 21-Nov-2014 |
| Castilla y León | Official Gazette of Castilla y León no. 249 30-Dec-2013 |
| Catalonia | Official Gazette of the Autonomous Government of Catalonia no. 6387 31- May-2013 |
| Extremadura | Official Gazette of Extremadura no. 35 19-Feb-2018 |
| Galicia | Official Gazette of Galicia no. 96 21-May-2014 |
| La Rioja | Official Gazette of La Rioja no. 156 19-Dec-2014 |
| Madrid | Official Gazette of the Community of Madrid no. 198 7-Aug-2017 |
| Murcia | Official Gazette of Murcia no. 48 28-Feb-2017 |
| Navarra | Official Gazette of Navarra no. 14 22-Jan-2019 |
| Basque Country | <https://www.osakidetza.euskadi.eus/contenidos/informacion/osk_servic_para_empresas/es_def/adjuntos/LIBRO-DE-TARIFAS_2020_osakidetza.pdf> |
| Valencia | DOGV no. 8202 30-Dec-2017 Fees Act Valencian Community - Health system fees (page 49735) |

Supplementary Table 2 Resource use for patients with angina

|  | Year 1 follow-up (n = 6,794) | | Year 2 follow-up (n = 6,550) | |
| --- | --- | --- | --- | --- |
|  | % of patients* |  per patient^**^ | % of patients* |  per patient^**^ |
| Visits and hospital stays | | | | |
| Primary care visits | 100.0 | 15.1 | 100.0 | 13.3 |
| Outpatient department visits ^I^ | 88.5 | 3.8 | 88.3 | 3.0 |
| Emergency room | 69.6 | 1.2 | 63.8 | 1.0 |
| Days of hospital stay | 54.8 | 0.9 | 62.2 | 0.9 |
| Therapeutic procedures ^II^ | 17.3 | 2.7 | 14.1 | 2.4 |
| Diagnostic tests | | | | |
| Blood tests | 82.6 | 2.6 | 71.4 | 2.4 |
| Conventional X-ray | 29.9 | 0.5 | 24.7 | 0.7 |
| Computerized Axial Tomography | 57.5 | 0.8 | 56.3 | 0.8 |
| Magnetic Resonance Imaging | 57.1 | 0.7 | 33.7 | 0.5 |
| Other diagnostic procedures ^III^ | 71.3 | 3.2 | 53.5 | 2.4 |
| Productivity loss (Indirect costs) | | | | |
| Temporary disability (days off work) | 12.3 | 11.9 | 11.2 | 11.2 |
| Permanent disability | 1.0 | --- | 1.1 | --- |
| Premature mortality | 0.3 | --- | 0.4 | --- |

*^*^ Percentage of patients who use the resource within the total population; ^**^ Average use per patient within the total population; ^i^ Cardiology, vascular surgery, endocrinology, geriatrics, internal medicine, neurology and/or rehabilitation; ^II^ Catheterization, angioplasty, bypass, endarterectomy, thrombectomy and rehabilitation therapy; ^III^ Echocardiogram, stress test and Holter monitoring.*

Supplementary Table 3 Resource use for patients with acute myocardial infarction

|  | Year 1 follow-up (n = 5,556) | | Year 2 follow-up (n = 5,414) | |
| --- | --- | --- | --- | --- |
|  | % of patients* |  per patient^**^ | % of patients* |  per patient^**^ |
| Visits and hospital stays | | | | |
| Primary care visits | 100.0 | 12.8 | 100.0 | 11.0 |
| Outpatient department visits ^I^ | 89.6 | 3.9 | 88.2 | 2.9 |
| Emergency room | 62.7 | 1.0 | 58.7 | 0.9 |
| Days of hospital stay | 58.6 | 0.9 | 60.8 | 0.9 |
| Therapeutic procedures ^II^ | 13.6 | 2.1 | 11.2 | 1.6 |
| Diagnostic tests | | | | |
| Blood tests | 76.5 | 2.2 | 66.6 | 2.0 |
| Conventional X-ray | 23.7 | 0.4 | 19.8 | 0.6 |
| Computerized Axial Tomography | 55.7 | 0.8 | 53.6 | 0.7 |
| Magnetic Resonance Imaging | 57.0 | 0.7 | 31.6 | 0.5 |
| Other diagnostic procedures ^III^ | 70.8 | 3.0 | 51.6 | 2.3 |
| Productivity loss (Indirect costs) | | | | |
| Temporary disability (days off work) | 13.6 | 12.9 | 12.0 | 11.4 |
| Permanent disability | 1.1 | --- | 1.1 | --- |
| Premature mortality | 0.2 | --- | 0.4 | --- |

*^*^ Percentage of patients who use the resource within the total population; ^**^ Average use per patient within the total population; ^i^ Cardiology, vascular surgery, endocrinology, geriatrics, internal medicine, neurology and/or rehabilitation; ^II^ Catheterization, angioplasty, bypass, endarterectomy, thrombectomy and rehabilitation therapy; ^III^ Echocardiogram, stress test and Holter monitoring.*

Supplementary Table 4 Resource use for patients with ischemic stroke

|  | Year 1 follow-up (n = 6,388) | | Year 2 follow-up (n = 5,984) | |
| --- | --- | --- | --- | --- |
|  | % of patients* |  per patient^**^ | % of patients* |  per patient^**^ |
| Visits and hospital stays | | | | |
| Primary care visits | 100.0 | 14.9 | 100.0 | 13.0 |
| Outpatient department visits ^I^ | 87.0 | 3.1 | 86.3 | 2.7 |
| Emergency room | 69.0 | 1.1 | 64.2 | 1.0 |
| Days of hospital stay | 56.4 | 1.0 | 57.3 | 0.8 |
| Therapeutic procedures ^II^ | 10.6 | 1.6 | 8.5 | 1.4 |
| Diagnostic tests | | | | |
| Blood tests | 79.1 | 2.3 | 63.8 | 2.0 |
| Conventional X-ray | 20.7 | 0.3 | 17.1 | 0.5 |
| Computerized Axial Tomography | 54.6 | 0.7 | 53.5 | 0.7 |
| Magnetic Resonance Imaging | 60.5 | 0.7 | 28.7 | 0.4 |
| Other diagnostic procedures ^III^ | 74.1 | 3.2 | 54.7 | 2.4 |
| Productivity loss (Indirect costs) | | | | |
| Temporary disability (days off work) | 12.1 | 11.3 | 8.0 | 9.2 |
| Permanent disability | 1.0 | --- | 1.1 | --- |
| Premature mortality | 0.5 | --- | 0.3 | --- |

*^*^ Percentage of patients who use the resource within the total population; ^**^ Average use per patient within the total population; ^i^ Cardiology, vascular surgery, endocrinology, geriatrics, internal medicine, neurology and/or rehabilitation; ^II^ Catheterization, angioplasty, bypass, endarterectomy, thrombectomy and rehabilitation therapy; ^III^ Echocardiogram, stress test and Holter monitoring.*

Supplementary Table 5 Resource use for patients with transient ischemic attack

|  | Year 1 follow-up (n = 2,184) | | Year 2 follow-up (n = 2,052) | |
| --- | --- | --- | --- | --- |
|  | % of patients* |  per patient^**^ | % of patients* |  per patient^**^ |
| Visits and hospital stays | | | | |
| Primary care visits | 100.0 | 15.0 | 100.0 | 14.5 |
| Outpatient department visits ^I^ | 87.4 | 3.0 | 86.4 | 2.6 |
| Emergency room | 70.1 | 1.2 | 63.3 | 1.0 |
| Days of hospital stay | 61.2 | 1.0 | 63.8 | 0.9 |
| Therapeutic procedures ^II^ | 10.2 | 1.6 | 10.4 | 2.0 |
| Diagnostic tests | | | | |
| Blood tests | 81.4 | 2.6 | 65.2 | 2.3 |
| Conventional X-ray | 24.3 | 0.4 | 21.7 | 0.7 |
| Computerized Axial Tomography | 48.5 | 0.6 | 49.4 | 0.7 |
| Magnetic Resonance Imaging | 56.9 | 0.7 | 28.8 | 0.5 |
| Other diagnostic procedures ^III^ | 68.0 | 3.2 | 51.6 | 2.5 |
| Productivity loss (Indirect costs) | | | | |
| Temporary disability (days off work) | 11.4 | 10.6 | 7.5 | 7.2 |
| Permanent disability | 0.6 | --- | 0.7 | --- |
| Premature mortality | 0.4 | --- | 0.2 | --- |

*^*^ Percentage of patients who use the resource within the total population; ^**^ Average use per patient within the total population; ^i^ Cardiology, vascular surgery, endocrinology, geriatrics, internal medicine, neurology and/or rehabilitation; ^II^ Catheterization, angioplasty, bypass, endarterectomy, thrombectomy and rehabilitation therapy; ^III^ Echocardiogram, stress test and Holter monitoring.*

Supplementary Table 6 Resource use for patients with peripheral arterial disease

|  | Year 1 follow-up (n = 6,054) | | Year 2 follow-up (n = 5,744) | |
| --- | --- | --- | --- | --- |
|  | % of patients* |  per patient^**^ | % of patients* |  per patient^**^ |
| Visits and hospital stays | | | | |
| Primary care visits | 100.0 | 16.5 | 100.0 | 14.5 |
| Outpatient department visits ^I^ | 80.7 | 2.1 | 87.9 | 2.6 |
| Emergency room | 70.4 | 1.1 | 64.8 | 1.0 |
| Days of hospital stay | 59.9 | 1.0 | 63.8 | 0.9 |
| Therapeutic procedures ^II^ | 14.4 | 2.1 | 12.3 | 2.0 |
| Diagnostic tests | | | | |
| Blood tests | 81.7 | 2.6 | 69.0 | 2.3 |
| Conventional X-ray | 28.7 | 0.5 | 23.6 | 0.7 |
| Computerized Axial Tomography | 51.3 | 0.7 | 49.6 | 0.7 |
| Magnetic Resonance Imaging | 61.3 | 0.7 | 33.2 | 0.5 |
| Other diagnostic procedures ^III^ | 69.5 | 3.0 | 53.7 | 2.5 |
| Productivity loss (Indirect costs) | | | | |
| Temporary disability (days off work) | 13.7 | 13.3 | 10.3 | 12.2 |
| Permanent disability | 1.5 | --- | 1.6 | --- |
| Premature mortality | 0.6 | --- | 0.6 | --- |

*^*^ Percentage of patients who use the resource within the total population; ^**^ Average use per patient within the total population; ^i^ Cardiology, vascular surgery, endocrinology, geriatrics, internal medicine, neurology and/or rehabilitation; ^II^ Catheterization, angioplasty, bypass, endarterectomy, thrombectomy and rehabilitation therapy; ^III^ Echocardiogram, stress test and Holter monitoring.*

Supplementary Table 7 Follow-up costs of patients with angina (mean [SD]) in the value of euros in 2021

|  | Year 1 (n =6,794) | Year 2 (n =6,550) |
| --- | --- | --- |
| Visits and hospital stays | | |
| Primary care visits | 740 (533) | 372 (280) |
| Outpatient department visits ^I^ | 386 (388) | 304 (364) |
| Emergency room | 297 (371) | 261 (292) |
| Days of hospital stay | 4,191 (8,265) | 4,157 (6,930) |
| Therapeutic procedures ^II^ | 1,702 (4,236) | 1,509 (4,211) |
| Diagnostic tests | | |
| Blood tests | 35 (30) | 32 (33) |
| Conventional X-ray | 11 (19) | 15 (32) |
| Computerized Axial Tomography | 203 (223) | 187 (208) |
| Magnetic Resonance Imaging | 218 (217) | 164 (261) |
| Other diagnostic procedures ^III^ | 489 (263) | 365 (222) |
| Drug treatments | 1,239 (574) | 1,199 (564) |
| Productivity loss (Indirect costs) | | |
| Temporary disability | 1,208 (3,728) | 1,130 (4,682) |
| Permanent disability | 404 (3,801) | 412 (3,877) |
| Premature mortality | 354 (8,000) | 735 (15,263) |

*^i^ cardiology, vascular surgery, endocrinology, geriatrics, internal medicine, neurology and/or rehabilitation; ^II^ catheterization, angioplasty, bypass, endarterectomy, thrombectomy and rehabilitation therapy; ^III^ echocardiogram, stress test and Holter monitoring.*

Supplementary Table 8 Follow-up costs of patients with acute myocardial infarction (mean [SD]) in the value of euros in 2021

|  | Year 1 (n =5,556) | Year 2 (n =5,414) |
| --- | --- | --- |
| Visits and hospital stays | | |
| Primary care visits | 629 (494) | 307 (253) |
| Outpatient department visits ^I^ | 393 (379) | 297 (336) |
| Emergency room | 253 (346) | 227 (262) |
| Days of hospital stay | 3,883 (7,025) | 3,805 (6,352) |
| Therapeutic procedures ^II^ | 1304 (3,779) | 1,003 (3,247) |
| Diagnostic tests | | |
| Blood tests | 30 (28) | 27 (30) |
| Conventional X-ray | 9 (17) | 12 (28) |
| Computerized Axial Tomography | 183 (204) | 171 (198) |
| Magnetic Resonance Imaging | 212 (209) | 147 (247) |
| Other diagnostic procedures ^III^ | 469 (250) | 348 (208) |
| Drug treatments | 1,315 (688) | 1,204 (663) |
| Productivity loss (Indirect costs) | | |
| Temporary disability | 1,306 (3,849) | 1,149 (4,569) |
| Permanent disability | 403 (3,796) | 411 (3,873) |
| Premature mortality | 252 (6,662) | 552 (11,613) |

*^i^ cardiology, vascular surgery, endocrinology, geriatrics, internal medicine, neurology and/or rehabilitation; ^II^ catheterization, angioplasty, bypass, endarterectomy, thrombectomy and rehabilitation therapy; ^III^ echocardiogram, stress test and Holter monitoring.*

Supplementary Table 9 Follow-up costs of patients with ischemic stroke (mean [SD]) in the value of euros in 2021

|  | Year 1 (n =6,388) | Year 2 (n =5,984) |
| --- | --- | --- |
| Visits and hospital stays | | |
| Primary care visits | 730 (520) | 362 (282) |
| Outpatient department visits ^I^ | 319 (345) | 270 (337) |
| Emergency room | 291 (378) | 256 (320) |
| Days of hospital stay | 4,278 (7,888) | 3,699 (6,372) |
| Therapeutic procedures ^II^ | 1,031 (3,916) | 849 (3,166) |
| Diagnostic tests | | |
| Blood tests | 31 (30) | 27 (31) |
| Conventional X-ray | 7 (16) | 10 (26) |
| Computerized Axial Tomography | 172 (196) | 161 (184) |
| Magnetic Resonance Imaging | 222 (204) | 128 (229) |
| Other diagnostic procedures ^III^ | 494 (253) | 375 (220) |
| Drug treatments | 1,202 (497) | 1,005 (398) |
| Productivity loss (Indirect costs) | | |
| Temporary disability | 1,147 (3,667) | 930 (4,448) |
| Permanent disability | 385 (3,709) | 392 (3,784) |
| Premature mortality | 685 (12420) | 622 (14,432) |

*^i^ cardiology, vascular surgery, endocrinology, geriatrics, internal medicine, neurology and/or rehabilitation; ^II^ catheterization, angioplasty, bypass, endarterectomy, thrombectomy and rehabilitation therapy; ^III^ echocardiogram, stress test and Holter monitoring.*

Supplementary Table 10 Follow-up costs of patients with transient ischemic attack (mean [SD]) in the value of euros in 2021

|  | Year 1 (n =2,184) | Year 2 (n =2,052) |
| --- | --- | --- |
| Visits and hospital stays | | |
| Primary care visits | 740 (496) | 370 (274) |
| Outpatient department visits ^I^ | 307 (342) | 278 (342) |
| Emergency room | 300 (360) | 248 (294) |
| Days of hospital stay | 4,454 (7822) | 3,978 (5,979) |
| Therapeutic procedures ^II^ | 994 (3,339) | 1,061 (3,611) |
| Diagnostic tests | | |
| Blood tests | 34 (31) | 28 (32) |
| Conventional X-ray | 9 (17) | 12 (28) |
| Computerized Axial Tomography | 155 (194) | 155 (189) |
| Magnetic Resonance Imaging | 209 (203) | 138 (246) |
| Other diagnostic procedures ^III^ | 496 (260) | 369 (229) |
| Drug treatments | 1,204 (486) | 1,007 (385) |
| Productivity loss (Indirect costs) | | |
| Temporary disability | 1,072 (8,839) | 729 (3,752) |
| Permanent disability | 265 (3,082) | 270 (3,144) |
| Premature mortality | 246 (4,597) | 155 (3,960) |

*^i^ cardiology, vascular surgery, endocrinology, geriatrics, internal medicine, neurology and/or rehabilitation; ^II^ catheterization, angioplasty, bypass, endarterectomy, thrombectomy and rehabilitation therapy; ^III^ echocardiogram, stress test and Holter monitoring.*

Supplementary Table 11 Follow-up costs of patients with peripheral arterial disease (mean [SD]) in the value of euros in 2021

|  | Year 1 (n =6,054) | Year 2 (n =5,744) |
| --- | --- | --- |
| Visits and hospital stays | | |
| Primary care visits | 813 (635) | 405 (339) |
| Outpatient department visits ^I^ | 217 (280) | 265 (322) |
| Emergency room | 291 (370) | 258 (299) |
| Days of hospital stay | 4,339 (7,625) | 4,123 (6,411) |
| Therapeutic procedures ^II^ | 1,297 (3,619) | 1,226 (3,715) |
| Diagnostic tests | | |
| Blood tests | 35 (31) | 31 (33) |
| Conventional X-ray | 11 (19) | 14 (30) |
| Computerized Axial Tomography | 175 (210) | 164 (201) |
| Magnetic Resonance Imaging | 230 (209) | 157 (254) |
| Other diagnostic procedures ^III^ | 469 (254) | 379 (230) |
| Drug treatments | 1,207 (607) | 1,076 (559) |
| Productivity loss (Indirect costs) | | |
| Temporary disability | 1,341 (3,860) | 1,236 (5,200) |
| Permanent disability | 561 (4,468) | 572 (4,558) |
| Premature mortality | 761 (16,324) | 607 (11,324) |

*^i^ cardiology, vascular surgery, endocrinology, geriatrics, internal medicine, neurology and/or rehabilitation; ^II^ catheterization, angioplasty, bypass, endarterectomy, thrombectomy and rehabilitation therapy; ^III^ echocardiogram, stress test and Holter monitoring.*
